# Spatial limits of visuotactile interactions in the presence and absence of tactile stimulation

**DOI:** 10.1007/s00221-017-4998-0

**Published:** 2017-05-30

**Authors:** Laura Mirams, Ellen Poliakoff, Donna M. Lloyd

**Affiliations:** 1 School of Natural Sciences and Psychology, Liverpool John Moores University, Tom Reilly Building, Byrom Street, Liverpool, UK; 2 School of Psychological Sciences, University of Manchester, Manchester, UK; 3 School of Psychology, University of Leeds, Leeds, UK

**Keywords:** Vision, Touch, Somatic signal detection task, Rubber hand illusion, Peripersonal space, Reach space

## Abstract

The presence of a light flash near to the body not only increases the ability to detect a weak touch but also increases reports of feeling a weak touch that did not occur. The somatic signal detection task (SSDT) provides a behavioural marker by which to clarify the spatial extent of such visuotactile interactions in peripersonal space. Whilst previous evidence suggests a limit to the spatial extent over which visual input can distort the perception of tactile stimulation during the rubber hand illusion, the spatial boundaries of light-induced tactile sensations are not known. In a repeated measures design, 41 participants completed the SSDT with the light positioned 1 cm (near), 17.5 cm (mid) or 40 cm (far) from the tactile stimulation. In the far condition, the light did not affect hit, or false alarm rates during the SSDT. In the near and mid conditions, the light significantly increased hit rates and led to a more liberal response criterion, that is, participants reported feeling the touch more often regardless of whether or not it actually occurred. Our results demonstrate a spatial boundary over which visual input influences veridical and non-veridical touch perception during the SSDT, and provide further behavioural evidence to show that the boundaries of the receptive fields of visuotactile neurons may be limited to reach space.

## Introduction

Our perception of touch not only depends on the presence and nature of tactile stimulation to the body surface, but it can also be influenced by information from other sensory modalities. For example, when a light flashes in close proximity to the body, people are faster to detect tactile targets at the same body location (e.g. Butter et al. 1989). When vision and touch provide conflicting information, however, the visual system can dominate and alter touch perception (Johnson et al. 2006). During the rubber hand illusion (RHI; Botvinick and Cohen 1998), when a fake hand is positioned close to our body, and touched at the same time as our real hand is touched (but is out of sight), it can result in the feeling that the touch is coming from the fake hand. Similarly, during the somatic signal detection task (SSDT; Lloyd et al. 2008), presenting a light next to the body increases “hits” (correct reports of feeling a weak touch) but also increases “false alarms” (false reports of feeling touch). These tactile illusions demonstrate the potential for visual stimulation, occurring in close proximity to the body, to distort the perception of touch. However, there are limits to the spatial extent over which touch can be referred from the real to the fake hand during the RHI (Lloyd 2007). The purpose of the present study was to determine the spatial boundary over which visual input influences both veridical and non-veridical touch perception, to further our understanding of the limits of visually evoked touch and peripersonal space.

Peripersonal space encompasses the space within reaching distance of the body, and is distinguished from personal space (the space directly on the body surface) and extrapersonal space (the area outside of reaching distance; Colby and Duhamel 1996; Colby 1998; Previc 1998). The extent of peripersonal space has been defined based on the response properties of bimodal neurons in the premotor and parietal cortices that are responsive to tactile stimulation on the surface of the body as well as visual stimulation in the area surrounding the body (see Graziano et al. 2004). Evidence from functional magnetic resonance imaging (fMRI) and behavioural studies suggests that similar multisensory representations of peripersonal space exist in humans (e.g. Lloyd et al. 2003; Làdavas and Farnè 2004; Makin et al. 2007).

Although the visual receptive fields of bimodal neurons are partially bound to the space surrounding the tactile receptive field on the body surface, always remaining within reaching space of the monkey (Fogassi and Gallese 2004), they are flexible. They extend with tool use (Iriki et al. 1996; Farnè et al. 2005; Bassolino et al. 2010) and are responsive when we see touch on a fake hand during the RHI (e.g. Ehrsson et al. 2004, 2005; Lloyd et al. 2006). Nevertheless, evidence from animal studies suggests that these neurons respond more strongly for visual stimuli positioned close to the body (within peripersonal space) as opposed to far from the body (Fogassi et al. 1996; Rizzolatti et al. 2002) and behavioural evidence is consistent with this. Studies with human participants have found stronger visuotactile interactions when a visual stimulus occurs near, as opposed to far from the body. For example, when a distracting light is presented at the same versus opposite location as a touch (at the finger versus the thumb), people are faster and more accurate in judging the location of the touch (Spence et al. 2004b). This effect is reduced when the distance between the tactile and visual stimulation is increased (e.g. Pavani and Castiello 2004; Spence et al. 2004a). These studies only compared visuotactile interactions in two distance conditions, however, with the visual stimulus in the ‘far’ conditions often being positioned next to a different (unstimulated) part of the body, rather than in extrapersonal space.

Behavioural paradigms, such as the RHI, have been used as proxy measures to investigate the neural representation, and spatial extent of peripersonal space (for a review see Makin et al. 2008). Lloyd (2007) positioned a fake right hand at six distances from the participant’s real right hand, from 17.5 cm to the left of the participant’s real hand (in line with their right shoulder), to 67.5 cm to the left of the participant’s real hand (across the body midline, at the limit of reach space for the right hand). Participants experienced the RHI (that is, agreed with the statement “it seemed as though the touch I felt was caused by the experimenter touching the rubber hand”), up to a distance of 27.5 cm; beyond this distance, the strength of the RHI diminished, and the time taken to elicit the illusion increased. This result may reflect the spatial limits of peripersonal space surrounding the hand. Beyond ‘reach space’ (which was approximately 30 cm in this study) visual input may no longer have the potential to distort touch perception, perhaps due to the limits of the visual receptive fields surrounding the hand.

Zopf et al. (2010) also investigated the effect of increasing the distance between the real and fake hands during the RHI. In their study, a fake left hand was positioned in the same location in front of the participant (close to the body midline), but the real hand (placed behind a screen) was positioned either 15, or 45 cm to the left of the fake hand. Whereas in Lloyd’s (2007) study, the visual information (seen position of the fake hand) differed between conditions, in Zopf et al.’s study, proprioceptive information (felt location of the real hand) differed between conditions. Zopf et al. (2010) found no difference in illusion strength between the two distance conditions and argued that a similar re-coding of peripersonal space towards the seen fake hand occurred in both distance conditions. In Zopf et al.’s study, the fake hand was always placed within reaching distance of the participants, but in Lloyd’s study, the fake hand was positioned at the limit of reach space. This may suggest that the occurrence of visual stimulation within reach space is required for vision to distort touch perception. An alternative explanation for Lloyd’s results, however, is that rotational differences between the real and fake hands accounted for the reduction in illusion strength, rather than the distance (Zopf et al. 2010). In Lloyd’s study, the fake hand was rotated to different degrees in each distance condition. Previous studies have found that rotational differences between the real and fake hands decrease the RHI (Pavani et al. 2000; Tsakiris and Haggard 2005; Holmes et al. 2006; Costantini and Haggard 2007). In the current study, we attempted to replicate Lloyd’s (2007) findings using a different paradigm, the somatic signal detection task (SSDT; Lloyd et al. 2008), which eliminated this distance/rotation confound and enabled us to also explore the effect of distance on light-evoked touch (i.e. false reports of feeling a touch).

During the SSDT, participants are asked to detect a weak vibration presented to their fingertip, which occurs on 50% of trials. When a nearby light (positioned 1.5 cm from the fingertip) flashes, participants make more “hits” but also more “false alarms” (reporting feeling the touch when it did not occur). As a result, there are small increases in sensitivity, but larger changes in response criterion in the presence of the light, that is, participants are more likely to report feeling the touch, regardless of whether it was presented (Lloyd et al. 2008; Mirams et al. 2012, 2013). As the strength of the vibration is at threshold (detectable on ~50% of trials), the presence of touch is ambiguous, and participants are unaware of whether or not their experience of a tactile sensation is ‘true’, or ‘false’. Therefore, performance on this task is less subject to demand characteristics compared to the RHI, during which participants are aware of experiencing the illusion. Furthermore, the SSDT enabled us to investigate the spatial limits of the influence of a visual stimulus on veridical (true) and non-veridical (false) touch perception, within the same paradigm.

The light may influence the detection of touch during the SSDT because it occurs within the tactile receptive field surrounding the participant’s hand, i.e. within the boundaries of peripersonal space. We have previously found that the light influences touch perception during the SSDT even when the hand is covered (but the nearby light still visible; Mirams et al. 2010), which is consistent with the neurophysiological evidence that visuotactile neurons respond to nearby visual stimulation even when the body part is covered (e.g. Graziano et al. 1997; Obayashi et al. 2000). The distance between the light and the body during the SSDT has been separated in one previous study. Durlik et al. (2014) presented tactile stimulation to the face, and the light flash approximately 1 m in front of the participant, at eye level. Durlik et al. still found a significant effect of the light on tactile sensitivity, hit rates, response criterion and false alarm rates. However, light-induced false alarm rates were much lower in their study (~4%), compared to the original SSDT paradigm, in which light-induced false alarm rates are around 10–15%. Indeed, other evidence suggests that spatial correspondence is not always necessary for multisensory integration to occur. Spence (2013) reviewed the evidence for the importance of spatial coincidence for multisensory integration, and argued that spatial correspondence is only crucial when a task involves a spatial component (i.e. discriminating the location of a target), but is less important for tasks which involve temporal judgements, or the simple detection of a target. If so, we may not expect increased distance to reduce the effect of the light during the SSDT. In the current study, the distance between the hand and the light was varied in a within-subjects design to provide an alternative behavioural marker of the response properties of visuotactile neurons encoding peripersonal space around the hand. Following Lloyd’s (2007) findings, the effect of the light was expected to be reduced when the light was positioned more than 30 cm from the participant’s hand. We also investigated potential distance condition order effects, given that participants would have more or less experience of the light-touch contingency during the SSDT depending on which distance condition they completed first. Although increased experience of the light-touch contingency during the SSDT does not seem to increase the effect of the light (McKenzie et al. 2010, 2012), a training protocol to reduce the light-touch association has been found to decrease false alarms during the SSDT (McKenzie et al. 2012). Therefore, distance condition order was counterbalanced and included as a covariate in our analyses.

## Materials and methods

### Participants

Forty-one right-handed participants aged 18–51 years (*M* = 24.61 years, SD = 6.71, 27 female) took part. Handedness was assessed using the Edinburgh handedness inventory (Oldfield 1971). All participants had normal or corrected to normal vision, and none reported any tactile sensory deficits. The study was approved by the Liverpool John Moores University (LJMU) Research Ethics Committee. Participants were recruited via poster advertisements and an online participation scheme website at LJMU. Informed consent was obtained from all individual participants included in the study.

### Study design

We used a 2 (light: present/absent) × 2 (touch: present/absent) × 3 (light-hand distance: 1 cm/17.5 cm/40 cm) repeated measures design. These three distance conditions are subsequently referred to as the near, mid and far conditions, respectively.

### SSDT materials

**Fig. 1:**
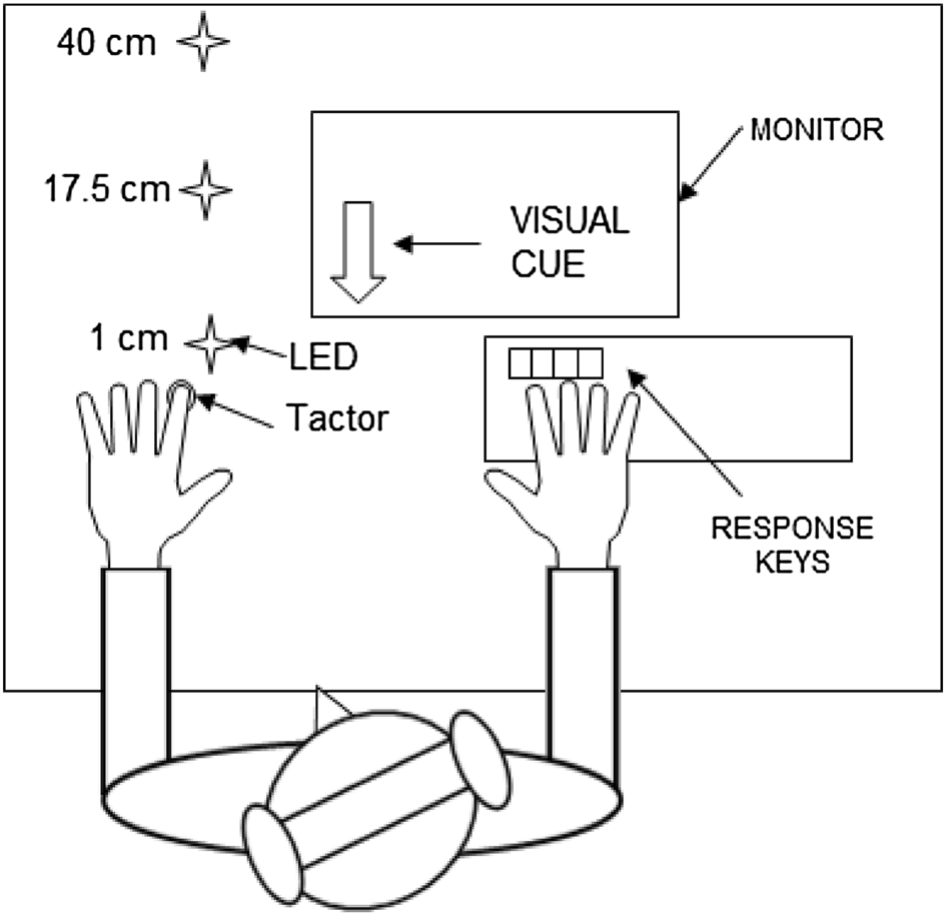
An illustration of the experimental setup

Participants sat in a light-attenuated room approximately 60 cm in front of a stimulus array. This consisted of a polystyrene block into which was mounted a tactor with a diameter of 18 mm (Dancer Design, St Helens, UK) and was attached to the underside of the participant’s fingertip with a double-sided adhesive pad. A 4 mm red light-emitting diode (LED) was mounted into the bottom left hand corner of a small black box, which could be positioned in one of three locations at increasing distances from the tactor (1, 17.5 or 40 cm). Tactile vibrations (20 ms, 100 Hz square wave vibrations) were produced by sending amplified sound files (using a Tactamp, Dancer Design), controlled via E-Prime software (Psychology Software Tools, Inc., Pittsburgh, PA, USA), to the tactor. Instructions were delivered on a computer monitor. Participants listened to white noise via headphones throughout the experiment to mask any sounds from the tactor (see Fig. 1 for an illustration of the experimental set-up).

### Thresholding procedure

Before beginning the main trials of the SSDT, a threshold was found for each participant using the parameter estimation by sequential testing (PEST; Taylor and Creelman 1967) algorithm, which is an adaptive method for quickly and efficiently estimating psychophysical parameters. The beginning of each trial was signalled by the appearance of a green arrow cue on the computer monitor (subtending approx. 18° × 7° of the visual angle) pointing towards the participant’s left index finger for 250 ms. This was followed by two stimulus periods of 1020 ms. In one of the time periods, the 20 ms tactile pulse was delivered with a delay of 500 ms on either side; in the other time period, an empty 1020 ms period occurred. An on-screen prompt then appeared, and participants were asked to report whether they had felt a pulse in the first or second time period by pressing the “1” or “2” key on the computer keyboard (a two alternative forced choice design). The PEST procedure began by presenting an above threshold vibration which was the same intensity for all participants. If participants responded correctly on a series of trials (>75% correct responses), the programme automatically reduced the strength of the vibration (by lowering the volume of the sound file used to produce the vibration). If they began to respond incorrectly (<75% correct), it increased the vibration strength. This procedure was repeated until the stimulus intensity approached the participant’s 75% threshold (the intensity necessary for participants to identify the correct time period in 75% of trials). A Wald (1947) sequential likelihood-ratio test was used to determine when to change the strength of the vibration. The thresholding procedure took approximately 5 min on average. Initial step size was set to 800 (as e-prime specifies volume in hundredths of decibels, this resulted in an initial decrease in the volume of the sound file equal to 8 dB). Subsequent step size was determined using the following rules:

1. On every reversal of direction, the step size is halved (unless it follows a double, see rule 3).
2. The second step in a given direction is the same size as the first.
3. If a sequence of three steps in the same direction occurs, then double the step size.
4. The fourth and subsequent steps in the same direction are each double the step size of their predecessor.
5. After each reversal that follows a double, no change to the step size.
6. End when the minimum step size is reached (this was set to 50).

If the minimum step size was not reached after 150 trials, the vibration strength was set to the average stimulus strength over the last 50 thresholding trials (this was the case for five participants).

### SSDT design and procedure

The main blocks of the SSDT consisted of 80 trials with the following trial types: light only (light present/touch absent); light and touch (light present/touch present); touch only (light absent/touch present); and catch (light absent/touch absent) presented 20 times per block in a random order. The tactile stimulus was presented at the threshold level previously established. Each SSDT trial was preceded by the appearance of the green arrow cue on the monitor for 250 ms. In touch only trials, a 20 ms vibration was presented with a delay of 500 ms before and after. Catch trials consisted of an empty 1020 ms interval. In light and touch trials, the LED flashed for 20 ms at the same time as the vibration. In light only trials, the LED flashed for 20 ms alone. Participants were not told anything about the light and were only required to indicate whether or not they felt a touch after each trial by entering a number corresponding to one of four response options: “definitely yes” (1), “maybe yes” (2), “maybe no” (3), “definitely no” (4). Participants were instructed to keep their hand still throughout the experiment, including break and rest periods. Each participant completed the SSDT under the three hand-light distance conditions (80 trials per condition, 3 blocks of main trials in total) with a 1 min break between conditions. The order of conditions was counterbalanced between participants. In each condition, the black box containing the LED was positioned in one of the three distance locations and was moved by the experimenter during the break between each block. After the final block, participants completed the thresholding procedure for a second time to determine whether threshold levels remained stable. No other instructions were given and participants were naıve as to the true purpose of the study. The experiment lasted 50 min in total.

### Analysis

To calculate signal detection theory statistics *d*′ and *c* (Macmillan and Creelman 1991) responses were classified as hits (reports of feeling the touch on touch-present trials), misses (reports of not feeling the touch on touch-present trials), false alarms (reports of feeling the touch on touch-absent trials) or correct rejections (reports of not feeling the touch on touch-absent trials). Some participants did not use all of the response options in all light/distance conditions; therefore, ‘definitely’ and ‘maybe’ responses were combined and grouped into ‘yes’ and ‘no’ responses. Hit rates [(hits + .5)/(hits + misses + 1)] and false alarm rates [(false alarms + .5)/(false alarms + correct rejections + 1)] were calculated using the log-linear correction^1^ (Snodgrass and Corwin 1988). These were used to calculate the signal detection theory test statistics *d*′ [*z*(hit rate)−*z*(false alarm rate)] and *c* [−.5*z(hit rate) + *z*(false alarm rate)]. This provided estimates of each participant’s perceptual sensitivity (*d*′) and tendency to report stimuli as present (response criterion, *c*) in the light and distance conditions. Statistical analyses were conducted using SPSS version 15.0 (SPPS Inc., Chicago, IL).

## Results

### Descriptive statistics

Table 1 shows descriptive statistics for hit rates, false alarm rates, sensitivity (*d*′) and response criterion (*c*) in each SSDT light and light-hand distance condition. Threshold levels did not change significantly from the beginning (*M* stimulus level = −515.42, SD = 287.59) to the end of the testing session (*M* stimulus level = −593.98, SD = 344.33, *t* (40) = 1.48, *p* = .15). The false alarm rate data in each distance and light condition were significantly positively skewed. Log and square root transformations did not normalise the data; therefore, non-parametric tests were used to analyse these data.

**Table 1:** Descriptive statistics for hit rate, false alarm rate, *d*′ and *c* in each distance and light condition

		Hit rate	False alarm rate		*d*′	*c*
		*M* (SD)	*M* (SD)	Mdn (range)	*M* (SD)	*M* (SD)
NEAR	Light	52.32 (29.14)	14.92 (15.68)	7.14 (57.00)	1.32 (1.11)	.58 (.60)
	No light	48.61 (27.93)	12.95 (15.67)	7.14 (62.00)	1.35 (1.13)	.69 (.58)
MID	Light	51.28 (26.60)	12.37 (12.64)	7.14 (48.00)	1.38 (.95)	.63 (.53)
	No light	43.47 (27.79)	11.44 (13.12)	7.14 (71.00)	1.25 (1.09)	.77 (.53)
FAR	Light	47.68 (29.89)	12.49 (14.57)	7.14 (57.00)	1.32 (1.09)	.71 (.61)
	No light	43.84 (29.99)	11.56 (12.34)	7.14 (57.00)	1.20 (1.07)	.77 (.62)

### Hit rates

A 2 (light) × 3 (distance) ANOVA with distance condition order (near first, mid first, far first) as a covariate was conducted. The main effects of light (*F* (1, 39) = 1.32, *p* = .26) and distance (*F* (2, 78) = .01, *p* = .99) were not significant. However, there was a light × distance interaction (*F* (2, 78) = 4.39, *p* = .02) and a light × distance × condition order interaction (*F* (2, 78) = 4.43, *p* = .02). No other effects were significant (*p*’s ≥ .79).

To follow up these interactions, mixed design ANOVAs with light as a within-subjects factor and condition order as a between-subjects factor were conducted separately for each distance condition. In the near condition, there was a significant effect of the light (*F* (1, 38) = 4.15, *p* = .04) with a higher hit rate in the presence of the light, but no effect of condition order (*F* (2, 38) = .27, *p* = .76) and no light × condition order interaction (*F* (2, 38) = .43, *p* = .66). In the mid-condition, there was also a significant effect of the light (*F* (1, 38) = 12.81, *p* = .001), but no effect of condition order (*F* (2, 38) = .18, *p* = .83) and no light × condition order interaction (*F* (2, 38) = .18, *p* = .83). In the far condition, there was no longer a significant effect of the light (*F* (1, 38) = 3.32, *p* = .08), no effect of condition order (*F* (2, 38) = .65, *p* = .53) and no light × condition order interaction (*F* (2, 38) = 2.33, *p* = .11).

### False alarm rates

**Fig. 2:**
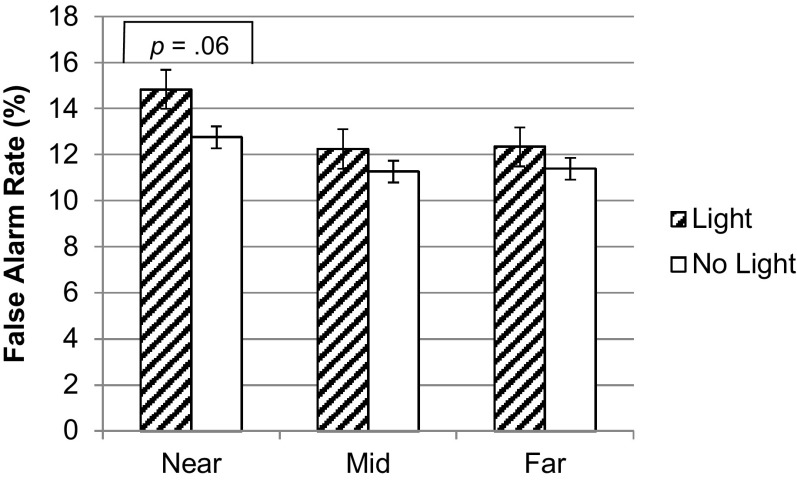
Mean false alarm rate in each light and distance condition

Separate Wilcoxon tests were conducted to investigate the effect of the light on false alarm rates in each distance condition (with the significance level lowered to *p* = .02 to correct for multiple comparisons). There was a tendency towards an effect of light in the near condition (*Z* = 1.87, *p* = .06) but no effect of the light in the mid (*Z* = .30, *p* = .77) or far (*Z* = .43, *p* = .66) conditions, see Fig. 2.

### Sensitivity (*d*′)

**Fig. 3:**
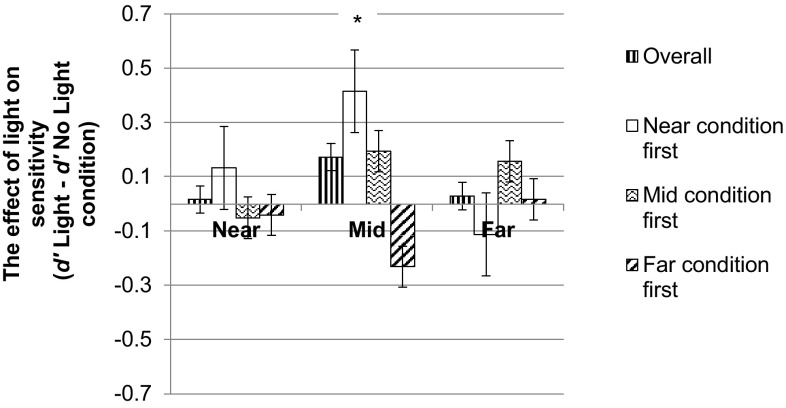
The effect of the light (*d*′ light–*d*′ no light) in each distance condition overall and in participants who did the near, mid and far conditions first. *Asterisk* the effect of the light was significant in the mid condition, only for participants who did the near condition first

A 2 (light) × 3 (distance) ANOVA with distance condition order (near first, mid first, far first) as a covariate was conducted. The main effects of distance and light were not significant (*F* (2, 78) = 1.65, *p* = .20, and *F* (1, 39) = 2.86, *p* = .10, respectively). There was, however, a distance × light interaction (*F* (2, 78) = 3.38, *p* = .04) and a distance × light × condition order interaction (*F* (2, 78) = 3.66, *p* = .03, see Fig. 3). No other effects were significant (*p*’s ≥ .10).

To follow up these interactions, mixed design ANOVAs with light as a within-subjects factor and condition order as a between-subjects factor were conducted separately for each distance condition. In the near condition, there was no effect of light (*F* (1, 38) = .02, *p* = .88) or condition order (*F* (2, 38) = .42, *p* = .66) and no light × condition order interaction (*F* (2, 38) = .78, *p* = .47, see Fig. 3). In the mid-condition, there was a tendency towards an effect of the light (*F* (1, 38) = 4.23, *p* = .05) and a light × condition order interaction (*F* (2, 38) = 3.94, *p* = .03). The main effect of condition order was not significant (*F* (2, 38) = .10, *p* = .91). For participants who did the near condition first, *d*′ was significantly higher in the light (*M* = 1.45) compared to the no light condition (*M* = 1.03, *t* (11) = 6.13, *p* < .001). For participants who did the mid and far conditions first, *d*′ was not significantly different in the light and no light conditions (*t* (16) = .57, *p* = .58, and *t* (11) = .57, *p* = .58, respectively, see Fig. 3). In the far condition, there was no effect of the light (*F* (1, 38) = 1.64, *p* = .21), no effect of condition order (*F* (2, 38) = 2.42, *p* = .10) and no light × condition order interaction (*F* (2, 38) = 2.15, *p* = .13, see Fig. 3). To summarise, the light affected *d*′ only in participants who did the near condition first. For these participants, the light had a significant effect on *d*′ in the mid condition only.

### Response criterion (*c*)

A 2 (light) × 3 (distance) ANOVA with condition order (near first, mid first, far first) as a covariate was conducted. There was no main effect of light (*F* (1, 39) = .00, *p* = .99), no main effect of distance (*F* (2, 78) = 1.28, *p* = .28) but a light × distance interaction (*F* (2, 78) = 3.20, *p* = .04). Paired samples *t* tests with the Bonferroni correction for multiple comparisons showed that response criterion was significantly lower in the light, than the no light condition in the near (*t* (40) = 2.51, *p* = .02) and mid (*t* (40) = 2.70, *p* = .01) conditions, but not in the far condition (*t* (40) = .94, *p* = .35).

### Results summary

There were no significant multisensory effects of the light on hit rates, false alarm rates, *d*′ and *c* in the far condition, while there were effects for the near and mid conditions. In the near condition, the light increased hit rates and tended to increase false alarm rates, leading to a significant change in bias towards reporting a touch, but no significant increase in sensitivity. In the mid-condition, the light significantly increased hit rates and shifted bias as well as increasing *d*′ for those participants who experienced the near condition first.

## Discussion

Whereas previous evidence suggests that visual input affects the perception of an existing touch to a greater extent when the visual input occurs close, as opposed to far from the body (Lloyd 2007), the spatial limits over which a visual stimulus has the potential to evoke the perception of touch are less clear. Altering the distance between the location of the tactile and visual input during the SSDT allowed us to get a clearer indication of the spatial limits over which a visual stimulus can affect the veridical perception of an existing touch, as well as the false perception of an absent touch. When the light was positioned next to the fingertip (1 cm) or within reaching distance of the participant (17.5 cm), we found significant effects of the light on hit rates, *d*′ and *c*. There was only a tendency for higher false alarm rates in the presence of the light in the near condition, but no effect of the light on false alarms in the mid or far conditions. As expected, beyond reaching distance (at 40 cm), the light no longer influenced the perception, or misperception of touch.

Our results are consistent with previous behavioural evidence that visuotactile interactions are stronger for visual stimuli occurring close, as opposed to far from the location of tactile stimulation (Spence et al. 2004b; Shore et al. 2006), perhaps due to the response properties of visuotactile neurons, which respond more strongly for visual stimuli positioned close to the body (e.g. Fogassi et al. 1996; Rizzolatti et al. 2002). In the current study, participants made more hit responses (detected more weak touches), in the presence of a light positioned 1 or 17.5 cm from their fingertip. At these hand-light distances, participants also had a more liberal response criterion in the presence of the light, being more likely to report feeling the touch regardless of whether or not it was presented. Our results suggest a division between peripersonal space and extrapersonal space. The effect of the light on hit rates and response criterion did not decrease along a near to far continuum. Instead, the effect of the light on hit rates and response criterion was of a similar magnitude in the near and mid conditions, in which the light was within reach space. This may reflect the limits of the visual receptive fields of bimodal neurons, which have been found to remain within reaching distance (Fogassi and Gallese 2004). Our results also suggest that the effect of the light on touch perception during the SSDT is due to spatial multisensory integration, rather than temporal integration (c.f. McKenzie et al. 2012) otherwise the light would have had the same effect in all conditions. Although Spence (2013) argued that spatial coincidence is only crucial in order for multisensory integration to occur when a task involves a spatial component, we found that some degree of spatial correspondence was necessary in order for visuotactile integration to occur, even though our task involved the simple detection of a tactile target.

According to signal detection theory (e.g. Macmillan and Creelman 1991), an individual decides whether or not a stimulus was presented depending on the strength of an internal decision signal, which is based on continuous output from the sensory system. Hits may have been higher in the presence of the light due to the activation of visuotactile neurons. Multisensory integration can produce neural activity greater than the sum of responses to two stimuli presented separately (e.g. Meredith and Stein 1986; Wallace et al. 1998). Therefore, the light may have boosted the strength of the internal decision signal when the touch was present (Pasalar et al. 2010), and also when the touch was absent, resulting in increased hits and a more liberal response criterion. Our finding that the light no longer increased hits at 40 cm (beyond reaching distance) provides further behavioural evidence to suggest that the boundaries of the receptive fields of visuotactile neurons may be limited to reach space (see Fogassi and Gallese 2004). To determine whether the limits of reach space do indeed account for the reduction in multisensory effects, hand-light distance could be manipulated differently in a future study, so that the light is always positioned in front of the participant, within reach space, but the hand is positioned at different lateral distances from the light, similar to Zopf et al.’s (2010) study.

In the current study, the light only tended to increase false alarms when it was presented 1 cm from the finger, although this effect was not significant, which contrasts with previous findings using this paradigm (e.g. Lloyd et al. 2008). Using the RHI paradigm, Lloyd (2007) found evidence to suggest that visual input can only distort the perception of touch when it occurs within reaching distance. In Lloyd’s study, self-reported experience of the RHI diminished when a fake hand was moved beyond 27.5 cm from the real hand. It seems that the spatial boundary over which visual input can elicit a false report of touch, as opposed to distort the perception of existing touch is even smaller, and perhaps limited to the area immediately surrounding the body. In future studies, it will be necessary to include additional distance conditions to determine whether a light does indeed have to occur right next to the body to elicit false reports of touch, or whether a light occurring between 1 and 17.5 cm could still elicit false alarms.

The lack of effect of the light on false alarms in the mid condition suggests that light-induced false alarms are driven by a different mechanism than the one responsible for increased hits in the presence of the light. Otherwise, we would expect the light to increase false alarms in the mid condition. Indeed, while it seems plausible that increases in the detection of touch in bimodal trials may be due to multisensory enhancement, light-induced false alarms cannot result from ‘bottom-up’ multisensory integration as only a single stimulus was presented (c.f. McKenzie et al. 2012). Alternatively, false alarms in the presence of the light may result from ‘top-down’ influences on perception. Vision can dominate and alter processing in other sensory modalities, particularly when the information from other sensory modalities is ambiguous (Johnson et al. 2006). Therefore, the light may be used to resolve uncertainty about the presence or absence of the vibration. This may be due to lifelong experience of a high correlation between spatially and temporally coincident multisensory events (c.f. Johnson et al. 2006). As a result, when the light flashes, we expect to feel a touch at the same time, but only when it flashes right next to our body, perhaps due to the association between seeing a stimulus approach the body and feeling a touch at the same time.

We did not find effects of the light on sensitivity in the near and far conditions. This result is consistent with previous findings from studies using the SSDT and a similar paradigm (Johnson et al. 2006; Lloyd et al. 2008; Mirams et al. 2012). In these studies, the nearby light led to small, non-significant increases in sensitivity, because increased hits in the presence of the light were accompanied by an increase in false alarms. In the mid condition, sensitivity was significantly higher in the presence of the light, but only for participants who did the ‘near’ condition first. This may have been because false alarms were reduced in the mid condition (for reasons discussed above). It is also possible that experiencing a spatial and temporal contingency between the light and touch in the near condition made it more likely that the light affected sensitivity when participants subsequently completed the mid condition. However, we have previously found that increased experience of the light-touch contingency during the SSDT does not increase the effect of the light (McKenzie et al. 2010, 2012). Alternatively, experiencing a lack of a spatial contingency between vision and touch (in participants who did the mid and far conditions first) could have made it less likely that the light affected sensitivity in subsequent blocks. McKenzie et al. (2012; experiment two) found that after a training protocol to reduce the association between the light and touch, participants made fewer false alarms during the SSDT (in both the presence and absence of the light). This suggests that experience of reduced light-touch contingency can indeed influence subsequent decision making during the SSDT. In McKenzie et al.’s study, the training protocol did not eliminate the effect of the light on hits, sensitivity and response criterion, perhaps due to lifelong learning of a strong association between spatially and temporally aligned visual and tactile stimuli.

The condition order effects apparent in the sensitivity data could also account for why the effect of the light on false alarms in the near condition did not quite reach significance in the current study. For the two-thirds of our participants who experienced a reduced spatial contingency between vision and touch (as a result of completing the mid and far conditions first), there may have been an overall reduction in false alarms during subsequent blocks of the task, which made it less likely to detect a significant effect of the light. Although a between-subjects design would have eliminated order effects, individual differences in the tendency to make false alarms (Brown et al. 2012) would have added variability to the data.

In our experiment, the light absent trials were physically equivalent in all distance conditions, but despite this, sensitivity in light absent trials was slightly higher in the near, followed by the mid and far conditions (see Table 1), although these differences were not significant.^2^ In light present trials, however, sensitivity was similar in each distance condition, despite these trials being physically different. We suggest that the slight increase in sensitivity in the near and mid conditions in light absent trials could have been due to increased attention to the spatial location of the hand, due to the presence of the nearby light on other trials within the block. In light present trials, this increased attention may not have resulted in higher sensitivity because of the concurrent increase in false alarms.

In summary, our results suggest a spatial boundary over which visual input influences touch perception during the SSDT, which seems to be limited to reach space. Our results contrast with Durlik et al.’s (2014) findings that the light affected all SSDT outcome measures when it was presented 1 m in front of the participant (with the tactile stimulation presented to the face). In Durlik et al.’s study, however, the light was positioned in the centre of the participant’s visual field throughout the experiment, which may have increased its salience. Furthermore, Durlik et al. did not compare different light-body locations, which limit comparison with the current results. Instead, we show that visuotactile integration no longer occurred when the light was positioned 40 cm from the location of touch, and an even higher degree of spatial correspondence seems to be necessary in order for a visual stimulus to increase false reports of touch, suggesting that we only expect a concurrent touch when visual stimulation occurs in close proximity to the body.

## Footnotes

1 Adding the values of 0.5 and 1 in the hit and false alarm rate formulas is recommended as it eliminates any false alarm rates of 0%, or hit rates of 100%, which causes problems for the subsequent calculation of sensitivity and response criterion.

2 Paired samples *t* tests suggested that *d*′ in light absent trials did not differ between the near and mid conditions (*t* (40) = .78, *p* = .44), the near and far conditions (*t* (40) = .95, *p* = .35), or the mid and far conditions (*t* (40) = .27, *p* = .79).
